# Achiasmate male meiosis in two *Cymatia* species (Hemiptera, Heteroptera, Corixidae)

**DOI:** 10.3897/zookeys.538.6722

**Published:** 2015-11-19

**Authors:** Desislava Stoianova, Snejana Grozeva, Nikolay Simov, Valentina Kuznetsova

**Affiliations:** 1 Institute of Biodiversity and Ecosystem Research, Bulgarian Academy of Sciences, 1 Tsar Osvoboditel, Sofia 1000, Bulgaria; 2 National museum of Natural History, Bulgarian Academy of Sciences, 1 Tsar Osvoboditel, Sofia 1000, Bulgaria; 3 Zoological Institute, Russian Academy of Sciences, Universitetskaya nab. 1, St. Petersburg 199034, Russia

**Keywords:** Karyotype, m-chromosomes, sex chromosome post-reduction, spermatocyte meiosis, morphology, Nepomorpha, Corixoidea

## Abstract

The karyotype and male meiosis, with a particular focus on the presence or absence of chiasmata between the homologs, were studied in the water boatman species *Cymatia rogenhoferi* (Fieber) and *Cymatia coleoptrata* (Fabricius) (Corixidae, Cymatiainae). It is shown that the species have 2n = 33 (28A+2m+X_1_X_2_Y) and 2n = 24 (20A+2m+XY) respectively, post-reduction of sex chromosomes, and achiasmate meiosis of an alignment type in males. Cytogenetic and some morphological diagnostic characters separating *Cymatia* Flor from the rest of Corixidae are discussed.

## Introduction

The Corixoidea, known as water boatmen, are moderately large to small aquatic insects, belonging to the true bug infraorder Nepomorpha. According to Schuh and Slater (1995), Corixoidea include the only family Corixidae, with six subfamilies: Corixinae, Cymatiainae, Diaprepocorinae, Heterocorixinae, Stenocorixinae, and Micronectinae. Nieser (2002a, b) raised Diaprepocorinae and Micronectinae to a family rank, meaning that Corixoidea is comprised of three families only: Corixidae, Micronectidae, and Diaprepocoridae. The validity of Nieser’s (2002b) system was criticised by Andersen and Weir (2004), but accepted by the majority of other authors (Tinerella 2008, Grozeva et al. 2008, Konopko et al. 2010, Weirauch and Schuh 2011, Fent et al. 2011). Chromosome data is currently available for Micronectidae as well as for the corixid subfamilies Corixinae and Cymatiainae. In Micronectidae all four hitherto studied species were reported to have achiasmate male meiosis and no m-chromosomes: three species of *Micronecta* Kirkaldy, 1897 display 2n = 24 (22A+XY), while one species of *Tenagobia* Bergroth, 1899 has 2n = 30 (28+XY) (Ituarte and Papeschi 2004, Grozeva et al. 2008). In Corixinae, all 30 studied has species from eight genera were shown to share common characteristics in males: chiasmate meiosis, sex chromosome post-reduction, presence of a pair of m-chromosomes, and karyotype with 2n = 24 (20+2m+XY) (Ueshima 1979, Waller and Angus 2005, Bressa and Papeschi 2007). Cymatiainae consist of two genera, *Cymatia* Flor, 1860 with dispersed Holarctic and Oriental distributions and the monotypic *Cnethocymatia* Jansson, 1982 from northern Australia and New Guinea (Štys and Jansson 1988). For the only studied species of Cymatiainae, *Cymatia bonsdorffi* (Sahlberg, 1819), the karyotype with 2n = 26 (24 + XY) was reported with no information on m-chromosomes and presence/absence of chiasmata in male meiosis (Slack 1938, Southwood and Leston 1959).

In meiosis, the chiasmata are known to tie homologous chromosomes together until their separation in the reductional division. However, in some animal groups, instead of chiasma formation, an achiasmate type of meiosis is observed, being, as a rule, restricted to the heterogametic sex (White 1973). In true bugs, when achiasmate meiosis presents, it seems to be stable and marks taxa at the rank of family (Grozeva et al. 2008a). Until the present time, this meiotic pattern has been found in seven families of Heteroptera, belonging to the infraorders Nepomorpha, Leptopodomorpha and Cimicomorpha (see Kuznetsova et al. 2011 for references).

In the present paper, the karyotype and male meiosis of other two *Cymatia* species, *Cymatia rogenhoferi* (Fieber, 1864) and *Cymatia coleoptrata* (Fabricius, 1777), were studied. The focal point of this work was to clarify the presence or absence of chiasmata in spermatocyte meiosis of these species.

## Material and methods

Five males of *Cymatia rogenhoferi* and two males of *Cymatia coleoptrata* were collected by light trap and hydrobiological net in different localities (Table 1). Males of *Cymatia coleoptrata* were fixed in 3:1 fixative (96% ethanol:glacial acetic acid mixture) in the field immediately after capturing. Males of *Cymatia rogenhoferi* were fixed in the field in 95% ethanol for subsequent sequencing, and the abdomen was transferred in 3:1 fixative for chromosome analysis, as it has been done recently by Nokkala et al. (2015) for *Cacopsylla myrtilli* (W. Wagner, 1947) (Psylloidea). The gonads were dissected out and squashed in a small drop of 45% acetic acid. The cover slip was removed using dry ice. Slides were dehydrated in fresh fixative (3:1) and air dried. The preparations were stained using Schiff-Giemsa method (Grozeva and Nokkala 1996).

**Table 1.** Material used for chromosome analysis

Species	Number of analysed males	Locality and date of collection
*Cymatia rogenhoferi*	5	Kazakhstan, Taukum Sands, near Topar River, eastern from Topar Village, 363m a. s. l., 45°02’12"N, 074°58’33"E, light trap, 31.05.2015, leg. N. Simov and F. Konstantinov
*Cymatia coleoptrata*	1	Bulgaria, Danube River, marsh Malak Preslavets, 20m a. s. l., 44°05'43"N, 026°50'23"E, 13.07.2014, leg. D. Stoianova
*Cymatia coleoptrata*	1	Bulgaria, Danube River, Srebarna lake, 13m a. s. l., 44°06'47"N, 027°03'34"E, 12.07.2014, leg. D. Stoianova

The chromosomes were analysed under light microscope (Axio Scope A1 – Carl Zeiss Microscope) at 100× magnification and documented with a ProgResMFcool – Jenoptik AG digital camera. All preparations and remains of the specimens are stored at the Institute of Biodiversity and Ecosystem Research (IBER), BAS in Sofia, Bulgaria.

## Results

### *Cymatia rogenhoferi*, 2n = 33 (28A+2m+X_1_X_2_Y)

**Figures 1–7.**
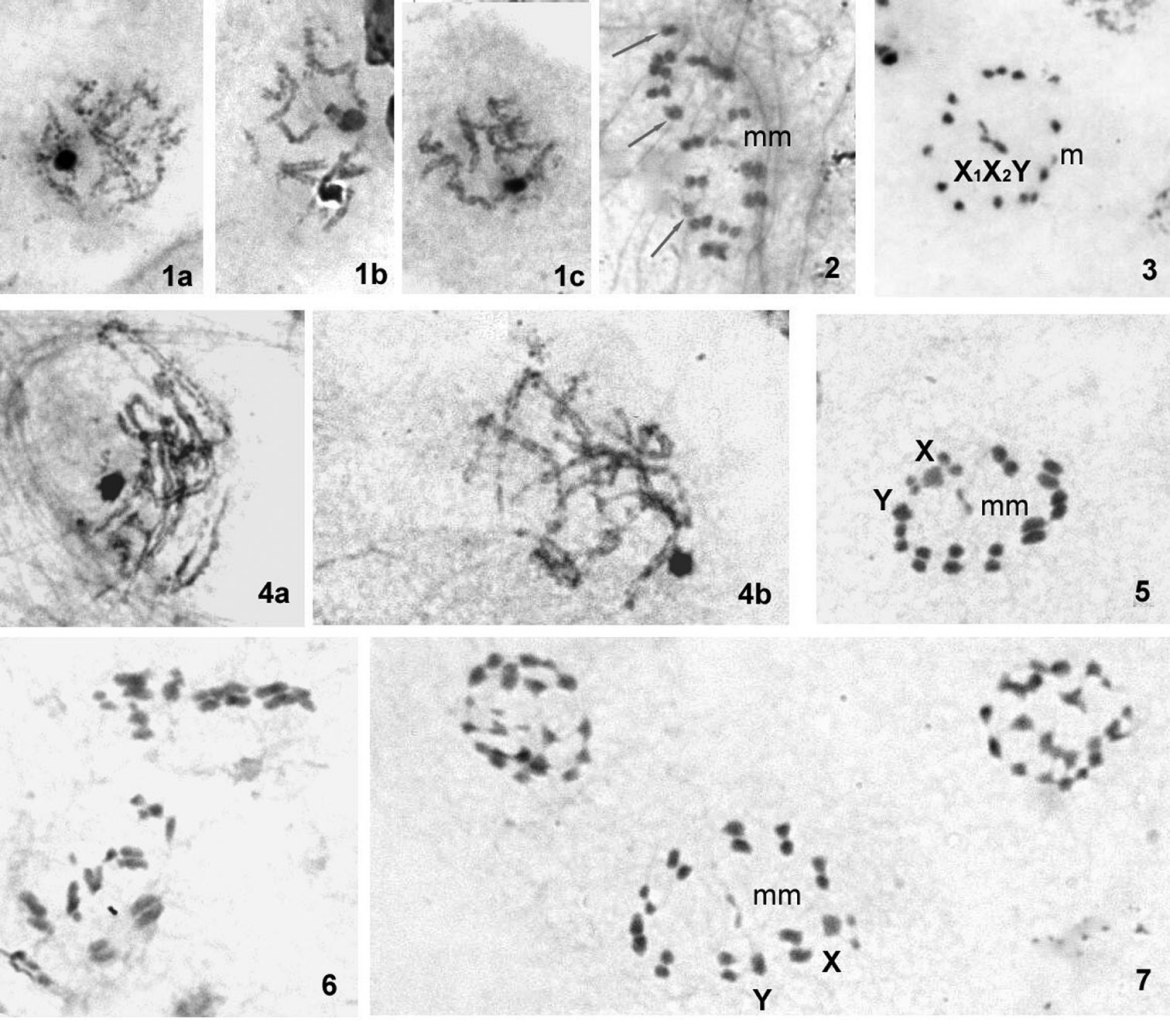
Male meiosis in *Cymatia* species. **1–3** *Cymatia rogenhoferi*: **a–c** early condensation stages **2** MI from the pole. The bivalents (consisting of two side-by-side aligned chromosomes facing the opposite poles) and three univalent sex chromosomes (two X and one Y) form a ring, with a pair of very small and negatively heteropycnotic m-chromosomes in its centre **3** MII. The autosomes and m-chromosome form a ring, with pseudo-trivalent of the sex chromosomes in its centre **4–7** *Cymatia coleoptrata*: **a, b** early condensation stages **5** MI from the pole. The bivalents (consisting of two side-by-side aligned chromosomes) and two univalent sex chromosomes (X and Y) form a ring, with a pair of very small and negatively heteropycnotic m-chromosomes in its centre **6** MI from the equator.The homologous autosomes can be seen lying parallel **7** late MI and AI plates. Scale bar = 10 µm.

The testes of the adult males were full of sperm, with a small number of well-synchronised dividing cells. No spermatogonial metaphases were observed. When condensing from a diffuse stage (Figs 1a, b, c), the autosomal bivalents consisted of side-by-side aligned homologous chromosomes without chiasmata, and the sex chromosomes appeared as a positively heteropycnotic body. No diplotene and diakinesis were present. At metaphase I (MI), the bivalents laid parallel to the equatorial plane, with the homologous chromosomes facing opposite poles without any sign of chiasmata. Clearly, male meiosis of this species is achiasmate. Both MI and MII were radial (Figs 2, 3). At MI, 14 autosomal bivalents and three univalent sex chromosomes (two X and one Y) formed a ring with, a pair of very small and negatively heteropycnotic m-chromosomes inside. In contrast to MI, the MII ring was formed by 14 autosomes and one m-chromosome, while the sex chromosomes formed a pseudo-trivalent placed inside the ring. The Y chromosome was clearly larger than each of the two X chromosomes (Fig. 3). The first division was thus reductional for the autosomes and m-chromosomes, but equational for the sex chromosomes (post-reduction). The chromosome formula of *Cymatia rogenhoferi* was determined as 2n = 33 (28A+2m+X_1_X_2_Y).

### *Cymatia coleoptrata*, 2n = 24 (20A+2m+XY)

The behaviour of chromosomes during the first spermatocyte division was quite similar to that in *Cymatia rogenhoferi*. Unfortunately, we found no second division stages in the two males explored here. When condensing from the diffuse stage (Figs 4a, b), the autosomal bivalents consisted of parallel aligned homologous chromosomes without traces of chiasmata, and the sex chromosomes appeared as a positively heteropycnotic body. No diplotene and diakinesis were observed. At metaphase I (MI), the bivalents were aligned parallel to the equatorial plane, with the homologous chromosomes facing opposite poles without any signs of chiasmata. The MI plates were radial (Fig. 5), with 10 autosomal bivalents and two univalent sex chromosomes (X and Y) forming a ring and a pair of very small and negatively heteropycnotic m-chromosomes placing inside it. The X chromosome was larger than the Y. Side by side association of homologous autosomes was still preserved at MI (Figs 5, 6) and anaphase I (AI) (Fig. 7), when the homologs moved in parallel to the opposite poles. Clearly, male meiosis of this species is achiasmate. The first division was reductional for the autosomes and m-chromosomes, but equational for the sex chromosomes (post-reduction).

The chromosome formula of *Cymatia coleoptrata* was determined as 2n = 24 (20A+2m+XY).

## Discussion

The main goal of this paper was to address the cytogenetic features of two species of *Cymatia* and compare them with those encountered within the superfamily Corixoidea. This entailed at least four related issues, namely: 1) whether the karyotypes are conservative in respect to chromosome number and sex chromosome system, 2) whether m-chromosomes are present, 3) whether the post-reduction of sex chromosomes is present, and 4) whether the chiasmata are formed in male meiosis. Both Corixidae and Micronectidae are known to be characterised by an XY sex chromosome system and an inverted sequence of X and Y chromosome divisions in spermatocyte meiosis, i.e. the sex chromosome post-reduction (for references see Ueshima 1979, Ituarte and Papeschi 2004, Waller and Angus 2005, Bressa and Papeschi 2007, Grozeva et al. 2008). Post-reduction means that the sex chromosomes behave as univalents during the first round of meiosis and undergo equational separation at AI whereas they form a pseudo-bivalent at MII and undergo reductional segregation at anaphase II. The species here studied, *Cymatia rogenhoferi* and *Cymatia coleoptrata*, were found to share the same characteristics with Corixidae and Micronectidae, i.e. an XY system and the sex chromosome post-reduction in male meiosis. Multiple X_1_X_2_Y mechanism found in *Cymatia rogenhoferi* might have originated by fragmentation of the initial X chromosome. The inverted sequence of sex chromosome divisions in spermatocyte meiosis is characteristic of the Heteroptera as a whole (Ueshima 1979), with rare exceptions (e.g. Golub et al. 2015). Other cytogenetic features, including chromosome numbers and presence or absence of m-chromosomes, whose origin, nature and significance are questionable (e.g. Nokkala 1986, Kuznetsova et al. 2011), and presence or absence of chiasmata in male meiosis, are distributed variously among different taxa of Corixidae and Micronectidae.

In Corixinae, each of 30 species studied display ten pairs of autosomes, a pair of very small m-chromosomes, and X and Y chromosomes: the karyotype formula of these species can be expressed as 2n = 24 (20A+2m+X+Y). Meiosis is of a standard chiasmate type in males (Ueshima 1979, Waller and Angus 2005, Bressa and Papeschi 2007). Compared to Corixinae, the family Micronectidae is less well studied. The karyotypes are currently known in Micronecta (Dichaetonecta) scholtzi (Fieber, 1860), Micronecta (Micronecta) poweri (Douglas & Scott, 1869), and Micronecta (Micronecta) griseola Horvath, 1899, each with 2n = 24 (22A+XY) (Grozeva et al. 2008), and in Tenagobia (Fuscagobia) fuscata (Stål, 1859), with 2n = 30 (28+XY) (Ituarte and Papeschi 2004). Based on the data available, Micronectidae differ from Corixinae in that they have alternative numbers of autosomes and no m-chromosomes. Furthermore, the species studied in Micronectidae show the achiasmate meiosis in males. *Cymatia rogenhoferi* and *Cymatia coleoptrata* studied in this paper, were found to have 2n = 33 (28A+2m+X_1_X_2_Y) and 2n = 24 (20A+2m+XY) respectively and achiasmate meiosis of an alignment type in males. In another *Cymatia* species, *Cymatia bonsdorffi* (Sahlberg, 1819), studied by Slack (1938) and later by Southwood and Leston (1959), the karyotype of 2n = 26 (24 + XY) was reported. Unfortunately, the authors provided no information on the special features of meiosis, including sex chromosomes’ behaviour. Thus, on the basis of the current state of knowledge, the Cymatiainae share a presence of m-chromosomes with Corixinae, while the absence of chiasmata is shared with Micronectidae. Due to their very small size and negative heteropycnosis during meiosis, m-chromosomes are easily overlooked by researchers, and subsequently information about the distribution of these puzzling structures in different true bug taxa can hardly be used for inferences.

The first (reductional in the majority of organisms) division involves several meiosis-specific events the most important being the formation of chiasmata, the points of genetic crossing-over, between homologous chromosomes. When meiosis is achiasmate and chiasmata are not formed, no diplotene or diakinesis stages can be recognised. The existence of achiasmate meiosis in phylogenetically unrelated true bug families, i.e. Micronectidae from the infraorder Nepomorpha (Ituarte and Papeschi 2004, Grozeva et al. 2008), Saldidae from the Leptopodomorpha (Nokkala and Nokkala 1983), and in several families of the Cimicomorpha (Nokkala and Nokkala 1984, Nokkala and Nokkala 1986a, b, Nokkala and Grozeva 2000, Grozeva and Nokkala 2002), argue for its repeated and independent origin in the evolution of Heteroptera. At the same time, the achiasmate meiosis in true bugs is probably of very ancient origins, since some divergence has occurred in its cytological characteristics during the evolution (Nokkala and Grozeva 2000, Grozeva et al. 2008). Consequently, true bugs evolved a diversity of achiasmate meiosis types that include a variety starting from an *alignment* type to a *colochore* type, including an intermediate type (Nokkala and Nokkala 1983, Nokkala and Nokkala 1984, Nokkala and Nokkala 1986a, b, Kuznetsova et al. 2007). Comprehensive classification of different types of achiasmate meiosis can be found in Kuznetsova et al. (2011). The most common type is achiasmate meiosis of the *alignment* type characterised by the tight side-by-side alignment of homologous chromosomes throughout prophase until MI. Meiosis of this type has been described in the Saldidae, Nabidae, Anthocoridae, Microphysidae, Corixoidea: Micronectidae (for references see Grozeva et al. 2008), and now also in another corixid group, the Cymatiainae (present study).

The Cymatiainae were erected for the first time as a separate taxon (as Cymatiini) in Corixidae on the basis of the shape and hairiness of the pala, the chitinisation of the pharynx, the length of maxillary stylets, and their position against pharynx (Walton in Hutchinson 1940). Later, the labium structure, the position of the labial sensilla, and absence of the strigil and stridulation mechanism, and the ability of sound production, as well as some other characters of the pala and embolium (Table 2) were added to the diagnosis (Jansson 1973, 1986, Schuh and Slater 1995, Nieser 2002b, Chen et al. 2005, Hädicke 2012, Broźek 2013a, b, 2014).

**Table 2.** Key diagnostic characters used to distinguish Cymatiainae from the rest of Corixidae

Cymatiainae	Corixidae
Labium without transverse sulcations	Labium with transverse sulcations
Absence of transverse pattern of distribution of the labial sensilla	Transverse pattern of distribution of the labial sensilla
Nodal furrow absent	Nodal furrow present
Pala elongate, nearly cylindrical in both sexes	Female pala spoon-shaped; male pala variable
Pala without pegs	Pala with pegs
Pala in both sexes without palm	Pala in both sexes with palm
Claw of hind leg inserted apically	Claw of hind leg inserted subapically
Absence of seta close to the claw’ basis	Presence of seta close to the claw’ basis
Strigil absent	Usually with strigil
Unable to stridulate	Stridulation by rubbing peg fields on the anterior femur against the side of the head, females of some species also able to stridulate
Achiasmate male meiosis	Chiasmate male meiosis

In different phylogenetic studies on Corixoidea (Zimmermann 1986, Mahner 1993, Hebsgaard et al. 2004, Hädicke 2012, Broźek 2014) the position of Cymatiainae varies from being considered a sister group of Corixidae *s. str.* (Corixinae + Heterocorixinae) or a basal taxon (together with Diaprepocoridae) in the whole superfamily Corixoidea. It has repeatedly been shown that the absence of chiasmata during spermatocyte meiosis is evolutionarily stable in true bugs, and marks taxa at the rank of family (for references see Grozeva et al. 2008, Kuznetsova et al. 2011). In this context, the finding of achasmate meiosis in Micronectidae (Grozeva et al. 2008) clearly supports the familial status of this group, earlier proposed by Nieser (2002a, b). Both achiasmate meiosis and a number of morphological diagnostic characters (Table 2) distinguish Cymatiainae from the rest of Corixidae. However, more comprehensive studies on morphological and cytogenetic aspects of Corixoidea as a whole are required to decide on the rank that should be assigned to Cymatiainae. The special focus must be on the genus *Cnethocymatia* from the same subfamily, the genus *Diaprepocoris* Kirkaldy, 1897 considered the most basal taxon of Corixoidea, and the genus *Stenocorixa* Horváth, 1926 showing morphological similarities with Cymatiainae (Hebsgaard et al. 2004, Hӓdicke 2012, Brożek 2014).
